# A method for automatically extracting infectious disease-related primers and probes from the literature

**DOI:** 10.1186/1471-2105-11-410

**Published:** 2010-08-03

**Authors:** Miguel García-Remesal, Alejandro Cuevas, Victoria López-Alonso, Guillermo López-Campos, Guillermo de la Calle, Diana de la Iglesia, David Pérez-Rey, José Crespo, Fernando Martín-Sánchez, Víctor Maojo

**Affiliations:** 1 Departamento de Inteligencia Artificial, Facultad de Informática, Universidad Politécnica de Madrid. Campus de Montegancedo S/N, 28660 Boadilla del Monte, Madrid, Spain; 2 Biomedical Informatics Group, Facultad de Informática, Universidad Politécnica de Madrid. Campus de Montegancedo S/N, 28660 Boadilla del Monte, Madrid, Spain; 3 Bioinformatics Unit, Institute of Health Carlos III, Carretera de Majadahonda a Pozuelo Km. 2, 28220 Majadahonda, Madrid, Spain; 4 Departamento de Lenguajes y Sistemas Informáticos, Facultad de Informática, Universidad Politécnica de Madrid. Campus de Montegancedo S/N, 28660 Boadilla del Monte, Madrid, Spain

## Abstract

**Background:**

Primer and probe sequences are the main components of nucleic acid-based detection systems. Biologists use primers and probes for different tasks, some related to the diagnosis and prescription of infectious diseases. The biological literature is the main information source for empirically validated primer and probe sequences. Therefore, it is becoming increasingly important for researchers to navigate this important information. In this paper, we present a four-phase method for extracting and annotating primer/probe sequences from the literature. These phases are: (1) convert each document into a tree of paper sections, (2) detect the candidate sequences using a set of finite state machine-based recognizers, (3) refine problem sequences using a rule-based expert system, and (4) annotate the extracted sequences with their related organism/gene information.

**Results:**

We tested our approach using a test set composed of 297 manuscripts. The extracted sequences and their organism/gene annotations were manually evaluated by a panel of molecular biologists. The results of the evaluation show that our approach is suitable for automatically extracting DNA sequences, achieving precision/recall rates of 97.98% and 95.77%, respectively. In addition, 76.66% of the detected sequences were correctly annotated with their organism name. The system also provided correct gene-related information for 46.18% of the sequences assigned a correct organism name.

**Conclusions:**

We believe that the proposed method can facilitate routine tasks for biomedical researchers using molecular methods to diagnose and prescribe different infectious diseases. In addition, the proposed method can be expanded to detect and extract other biological sequences from the literature. The extracted information can also be used to readily update available primer/probe databases or to create new databases from scratch.

## Background

Molecular technologies are used in routine clinical practice to identify microorganisms, and evaluate the presence of virulence factors, antibiotic resistance determinants and host-microbe interactions [1]. For instance, numerous nucleic acid assays have been developed [2] using hybridization or DNA extension techniques that include a wide range of technologies, such as polymerase chain reaction (PCR) methods [3], gene and whole genome sequencing [4,5], Luminex [6] and microarray analysis [7].

There is a wide range of technologies that provide specific short base sequences of DNA as probes — used to detect the complementary base sequence of interest—or as primers—that guide the DNA amplification process—used for different purposes. Primers and probes are the main components of nucleic acid-based detection systems and have been the subject of multiple studies. Therefore, different software programs have been developed to design these specific sequences of primers and probes minimizing potential cross-hybridization to be spotted, for example, as oligonucleotides in cDNA microarrays [8] or sequences of primers to amplify a segment of a unique target gene using reverse-transcriptase (RT)-PCR, or to identify a wide spectrum of human pathogens [9].

The biological literature is the main information source on probes and primers for infectious disease diagnosis and prescription. Primer and probe sequence data reported in the biomedical literature are an aid for the laborious task of primer and probe design for microorganism identification, genotyping and gene expression studies. Therefore, researchers need to search this information in the biomedical literature. Unfortunately, there are only a few online databases established as repositories for empirically validated primer and probe sequences submitted by researchers. These repositories include the Molecular Probe Data Base [10]—available through the Sequence Retrieval System [11]—which contains information on synthetic oligonucleotides used as primers or probes, or PrimerBank [12,13], created to retrieve primer information about humans and mice for gene expression analysis by PCR and Quantitative PCR (QPCR) [14]. Conversely, the NCBI Probe Database [15,16] is a public registry of nucleic acid reagents designed for use in a wide range of biomedical research applications. On the other hand, RTPrimerDB [17,18] and probeBase [19,20] are freely accessible databases containing empirically validated primer and probe sequences. All these resources are manually updated using the primer/probe information submitted by different researchers rather than automatically acquiring the sequences from the available literature.

Over the last few years, text mining, information extraction and knowledge engineering approaches have proven useful for automatically extracting, analyzing and visualizing biological information from the scientific literature for biomedical research [21-26]. Although text mining applied to biological data is an active research field, these techniques have not yet been applied to create methods and tools aimed at automating probe and primer extraction from scientific papers. Thus, to detect and identify target microorganisms and host-microbe interactions or design PCR and diagnostic microarrays, for instance, researchers normally have to manually review all the literature of interest to identify relevant primers and probes. This is a tough and time-consuming task.

In this paper we present an original method based on a combination of text mining, information extraction, and knowledge engineering techniques aimed at automatically detecting and extracting infectious disease-related primer and probe sequences from scientific papers. Given an input of a set of manuscripts in Adobe's Portable Document Format (PDF), our method returns the extracted primer/probe sequences that appear in the article annotated with information regarding their respective microorganism and gene-specific regions, if available

The paper is organized as follows. In the next section we describe the proposed method. Next, we present the results of the evaluation of the method using a test set of 297 papers containing 3999 sequences. After that, we discuss the results of the evaluation. Finally, we outline our conclusions in the last section.

## Methods

**Figure 1:**
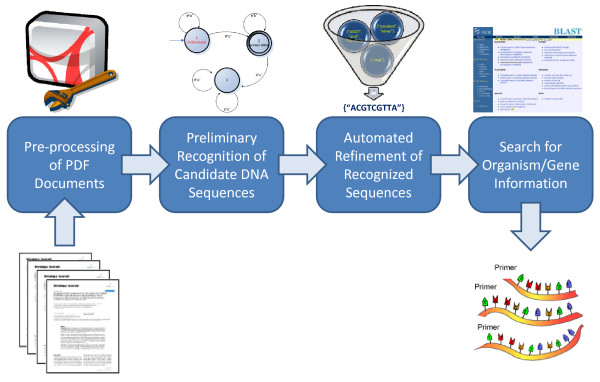
**Overview of the primer and probe extraction process**.

The proposed method for automatically extracting primer/probe sequences from the scientific literature is composed of four different activities. As shown in Figure 1, the method input is a set of manuscripts in PDF format, and its output is the set of extracted primer/probe sequences annotated with their respective organism/gene information, if available. Experts suggested working with PDF files since currently the PDF format is a *de facto *standard for publishing, archiving and exchanging electronic articles. However, to broaden the scope of the implementation of the proposed method, the software tool optionally accepts two additional input formats: PubMed Central XML and plain text.

The first activity of the method is aimed at converting the articles into a suitable format for applying text mining techniques. The objective of the second phase is to analyze the manuscript text and extract all the candidate sequences. They are filtered, refined and post-processed during the third activity. Finally, during the last phase, we query different public online genomic databases to automatically annotate the extracted sequences with their associated organism/gene information. A detailed description of each phase follows.

### Phase 1: Pre-processing of Articles

The pre-processing phase is aimed at translating each paper into a section tree (ST). The ST is a data structure that represents the structure of a document—i.e. sections and subsections—and stores its textual contents hierarchically.

STs are automatically built from the PDF files using a software tool developed by the authors. To build the ST, the software tool resorts to custom XML templates each describing the structure and layout of papers published in a particular journal or set of journals sharing a common layout—e.g. publications edited by BioMed Central.

To evaluate our method, we have focused on papers published in PDF format by several BioMed Central and PLoS journals since (1) our method requires full-text articles rather than abstracts to extract relevant information and BMC and PLoS papers can be freely accessed and downloaded, (2) different PLoS and BMC journals publish papers that are relevant for extracting information regarding infectious disease-related PCR primers and probes, and (3) most journals published by the same editorial group—either BMC or PLoS—share a common layout. Nevertheless, papers published by other journals can be easily converted into STs by creating a custom XML template to be used by the software tool for ST generation. Both the Document Type Definition (DTD) and the custom XML templates that we created for BMC and PLoS journals—which can be used as a guide to create custom templates for other journals—can be freely downloaded as part of the source code package at the project's homepage.

**Figure 2:**
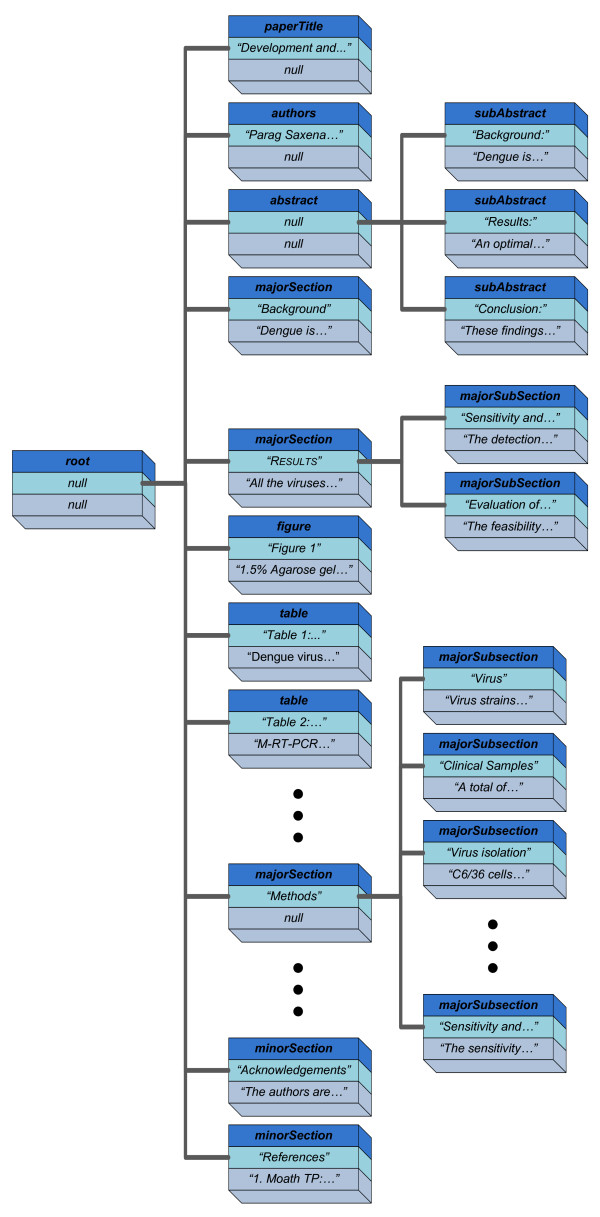
**ST corresponding to a PDF paper from the Virology Journal identified by PubMed ID 18234069**. Documents are organized hierarchically. The root node <***root***, *null*, *null*> represents the entire document. Complex sections (e.g. containing multiple subsections) are hierarchically decomposed according to the original paper structure. For instance, the section <***abstract***, *"Abstract"*, *null*> can be decomposed into its three child sections: <***subAbstract***, *"Background"*, *"Dengue is..."*>, <***subAbstract***, *"Results"*, *"An optimal..."*> and <***subAbstract***, *"Conclusion"*, *"These findings..."*>. Nodes of types ***table ***(e.g. <***table***, *"Table 2: Comparison of..."*, *"M-RT-PCR\tVirus isolation\nPositive\t96 (15.48%)\t..."*>) and ***figure ***(e.g. <***figure***, *"Figure 1"*, *"1.5% Agarose gel electrophoresis..."*>) are considered as special sections and thus allocated as children of the root node—the escape sequences "\t" and "\n" denote tab and newline characters. The natural reading order of the PDF paper can be reproduced by iterating the ST in depth-first order.

Figure 2 shows the ST for a sample PDF article published by the *Virology Journal*. As shown in Figure 2, each node represents a single section of the document, while parent-children links represent meronymy relationships. Thus, the different subsections represented by a set of child nodes are all subsections subsumed into a bigger section represented by the parent node. Regarding the structure of ST nodes, each contains a register-type data structure that records three different pieces of information. As shown in Figure 2, ST nodes are represented by tuples <***section_type***, *section_title*, *section_text*>, where ***section_type ***denotes the type assigned by our tool to the target section (i.e. one of the following: ***paperTitle***, ***abstract***, ***subAbstract***, ***majorSection***, ***subMajorSection***, ***subSubMajorSection***, ***minorSection***, ***table ***and ***figure***), *section_title *is the title of the section as extracted from the PDF document by the software tool, and *section_text *is the textual content associated with the target section—if empty, it is represented as *null*. There are some special section types such as ***table ***and ***figure***, which are considered by our software tool as being independent, even though they are usually located within a definite section in the PDF file. Thus, they are classed in the ST as children of the root node. We implemented this behavior since, when manually inspecting the PDF documents, we realized that (1) some figures and tables were not necessarily located within the section in which they are referenced in the PDF file, probably on layout grounds, and (2) a table or figure may be referenced by multiple sections.

As primer and probe sequences are often presented in tables (and text inside tables is organized into cells rather than paragraphs) they need to be handled differently from other sections of the papers. To extract and organize the text from tables we follow the approach described next. Table cells are processed by the PDF extractor from left to right and top to bottom. The text belonging to the current cell is extracted and concatenated with the text corresponding to the previously explored cells using an artificial delimiter. The latter was specifically chosen to ensure that the recognizers used in phase 2 will not merge the contents of two consecutive cells.

As stated previously, the software tool implementing our method optionally accepts manuscripts in (1) PubMed Central structured XML and (2) plain text format. Building the ST for manuscripts in PubMed Central XML format is straightforward, since the XML file already provides the document structure—including both tables and figures—that can be easily converted to a ST. Conversely, if users choose to feed the method with plain text articles, then the sectioning of the document is skipped—i.e. the full text of the manuscript is stored into a single section (node) of the ST—and users will be warned that this may decrease the accuracy of the method. In addition, as the source code of the application is freely available, users can write their own format converters (e.g. HTML) by creating instances of the *Document *class (i.e. the Java class implementing a ST) and filling in the different sections with the corresponding text.

Once the ST has been created, it is possible to fully reproduce the logical reading order of the original document by iterating the ST in depth-first order. This data structure is the input to the second activity described next.

### Phase 2: Preliminary Recognition of Candidate DNA Sequences

The next step of our method aims to recognize and extract DNA sequences appearing in the text. To address this task, we analyzed 40 papers selected by domain experts that contained the most common representations for PCR primers and probes in scientific literature. We would like to remark that (1) the training and test sets were disjoint, and (2) we did not have access to the test set at this stage. As a result of this study, we found that primer and probe DNA sequences are composed of individuals belonging to a 30-symbol alphabet—including 15 uppercase letters and their lowercase counterparts—that we will refer to as ∑ from now onwards. Similarly, we will denote the set of all different strings composed of one or more symbols from ∑ as ∑^+^. The latter includes symbols for the four nucleotide bases Adenine (A), Cytosine (C), Guanine (G), Thymine (T), whereas the remaining individuals are wildcard characters that represent a single nucleotide chosen from two or more nucleotide symbols. See Table 1 for further details on the wildcard-to-nucleotide mappings.

**Table 1:** Equivalences between alphabet symbols and nucleotides

Alphabet Symbol	Permissible Nucleotides	Complement	Meaning
**A**	A	T	[A]denine

**B**	C | G | T	V	Any but Adenine

**C**	C	G	[C]ytosine

**D**	A | G | T	H	Any but Cytosine

**G**	G	C	[G]uanine

**H**	A | C | T	D	Any but Guanine

**K**	G | T	M	[K]eto

**M**	A | C	K	A[M]ino

**N**	A | C | G | T	N	A[N]y nucleotide

**R**	A | G	Y	Pu[R]ine

**S**	C | G	S	[S]trong (3 H-bonds)

**T**	T	A	[T]hymine

**V**	A | C | G	B	Any but Thymine

**W**	A | T	W	[W]eak (2 H-bonds)

**Y**	C | T	R	P[Y]rimidine

This table (adapted from http://www.geneinfinity.org/sp_nucsymbols.html) shows the mappings between wildcard symbols used to represent DNA sequences in scientific papers and their permissible nucleotide types.

Regarding the representation of primer and probe sequences in scientific articles, we found that they may be enclosed by the strings *5' *and *3' *(or by *3' *and *5'*). Other papers, by contrast, present the sequences immediately after the string *5' *(or *3'*), but they do not terminate the sequence with the string *3' *(or *5'*). In both cases, the occurrence of the "prime" symbol is optional, since there are several manuscripts where the sequences are enclosed by strings *5 *and *3 *(or *3 *and 5) rather than *5' *and *3' *(or *5' *and *3'*). Similarly, other articles include sequences that do not appear enclosed between the strings *5' *and *3' *(*3' *and *5'*), but, instead, are sub-divided into *n *groups of exactly three nucleotides—except for the last group, which may include less than 3 bases. On the other hand, other manuscripts include sequences that do not match any particular pattern apart from being composed of symbols from ∑, blanks, dashes, colons and newline characters.

**Figure 3:**
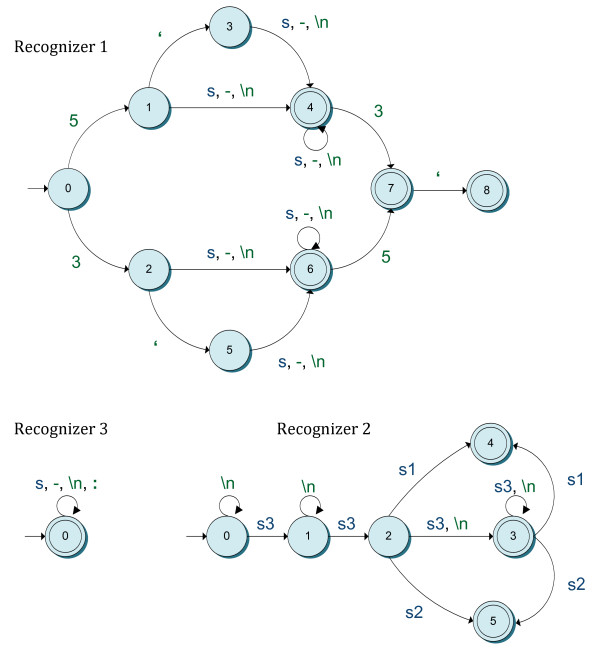
**State transition diagrams describing the preliminary sequence recognizers**. Circles represent regular states, whereas double circles stand for final (accepting) states. Edges denote state transitions triggered by the occurrence of any of the symbols drawn on the edges. These include 's' symbols in blue that represent strings of any length belonging to ∑^+^, whereas 's1', 's2' and 's3' are strings of symbols from ∑^+ ^of lengths 1, 2 and 3 respectively. Green items represent different literals such as dashes, colons, newline tokens, etc. States labeled with the number 0 that are pointed at by an arrow with no origin represent initial states.

Based on such findings, we created a set of three sequence detectors. The sequence detectors are applied to a textual input in a priority-based manner in decreasing order of specificity. If, given an input string, the current detector fails to match a sequence, then the input is forwarded to the next, more general recognizer. If a detector matches a sequence in an input string, confidence in the reliability of the matched sequence will be greater, the higher its priority is. In the following, we briefly describe the types of sequences detected by the different recognizers sorted by priority. (1) Sequences of symbols belonging to ∑ either delimited by the pairs of strings <*5'*, *3'*>, <*3'*, *5'*>, <*5*, *3*>, <*3*, *5*> or beginning with the strings *5'*, *5, 3'*, *3*. These sequences may also contain blanks, dashes and can span multiple lines. (2) Sequences organized into *n-1 *groups (with *n *> 2) of exactly three symbols from ∑ delimited by blanks plus an additional *n*^th ^group that may contain three or less symbols. These sequences can also span multiple lines. (3) Sequences of symbols from ∑ that can span several lines and that may also include blanks, colons and dashes. Table 2 presents some examples of nucleotide sequences that can be recognized by each detector. As shown in Figure 3, we followed a theoretical computer science approach to build the different recognizers using finite state machines [27].

**Table 2:** Sample sequences recognized by each detector

Detector	PMID	Text String	List of Tokens
**1**	19781080	...primers AA247 (5'-TGCCATTGCCAAAGAGAC-3') and pLQ510-rp1...	{"TGCCATTGCCAAAGAGAC"}

**1**	19664269	...mecA gene, mecAR (5'-TTACTCATGCCATACATAAATGGATA-\**n**GACG-3') and mecAF...	{"TTACTCATGCCATACATAAATGGATA", "GACG"}

**1**	19379498	...specific primer pair traD-F (5'-caatgcttgatctatttggtag-3') and traD-R...	{"caatgcttgatctatttggtag"}

**1**	19758438	...MY 09, 5-CGT CCM\**n **ARR GGA WAC TGA TC-3; where M = A/C, W = A/T...	{"CGT", "CCM", "ARR", "GGA", "WAC", "TGA", "TC"}

**2**	19799780	B-globin outside R @ CTC AAG TTC TCA GGA TCC A @ 1st round PCR primer for Human Beta globin DNA	{"CTC", "AAG", "TTC", "TCA", "GGA", "TCC", "A"}

**2**	18847469	btherm @ GAT GTG CCG GGC TCC TGC ATG @ This study	{"GAT", "GTG", "CCG", "GGC", "TCC", "TGC", "ATG"}

**2**	18154687	Stx1 @ GTA CGT CTT TAC TGA TGA TTG ATA GTG GCA CAG GG @ 35 @ 73.5	{"GTA", "CGT", "CTT", "TAC", "TGA", "TGA", "TTG", "ATA", "GTG", "GCA", "CAG", "GG"}

**2**	19558693	...are listed below.\n EP1- F ATG GTG GGC CAG CTT GTC\n EP1- R...	{"ATG", "GTG", "GGC", "CAG", "CTT", "GTC"}

**3**	19754958	...with primer N309 (ACATGCGGATCCCTCGAGCCTTTGAA-\nGATGACTAACTCCCCA) and N297...	{" ACATGCGGATCCCTCGAGCCTTTGAA", "GATGACTAACTCCCCA"}

**3**	19737401	...and 3' AAGCT TGGTA CCTCA CTGCA\nGCAGA GCGCT GAGGC CCAGC AGCAC. The resulting PCR...	{"AAGCT", "TGGTA", "CCTCA", "CTGCA", "GCAGA", "GCGCT", "GAGGC", "CCAGC", "AGCAC"}

**3**	19149882	1 @ XAC0340 @ 432 @ gATACCCCATATgAATgCgAT	{"gATACCCCATATgAATgCgAT"}

**3**	19775435	20 @ F:GAGATGGATTAACCAGATGTCTTAAAAACTATCGTAAC	{":","GAGATGGATTAACCAGATGTCTTAAAAACTATCGTAAC"}

This table shows some examples of sequences that can be recognized by each detector. The number under the column "Detector" identifies the detector that recognized the "List of Tokens" from the string "Text String" that can be found in the manuscript whose PubMed Identifier (PMID) is shown under the "PMID" column. The symbols **/n **and **@ **denote the newline and the table cell separator characters respectively.

To extract the candidate sequences occurring in a PDF paper, we feed the set of detectors with the text corresponding to the main sections of the manuscript—i.e. sections containing primer and probe sequences. These include sections whose type is one of the following: ***majorSection***, ***subMajorSection***, ***subSubMajorSection***, ***table ***and ***figure***. For instance, sections such as *"Background"*, *"Methods"*, *"Results"*, *"Discussion" *or *"Conclusions"—*all of type ***majorSection—***are provided as input to the detectors. Conversely, sections such as the paper title (***paperTitle***), authors (***authors***), *"Abstract" *(***abstract***), *"Acknowledgements" *(***minorSection***) or *"References" *(***minorSection***) are discarded. The text belonging to each section is obtained by iterating the ST associated with the manuscript being processed in depth-first order. Recognized sequences are then converted into a sequence of tokens composed of strings belonging to ∑^+^. Separator tokens—i.e. blanks, dashes and newline characters—are not included in the sequence, since (1) blanks are already implicit in the structure of the list of tokens and (2) both dashes and newline characters are no longer required for further processing. Conversely, colons are not discarded since they are required for the next stage. For instance, the string "5-CGT CCM\nARR-GGA-WAC-TGA" would be recognized by detector #1. This would produce the list of tokens {"CGT", "CCM", "ARR", "GGA", "WAC", "TGA"}. Table 2 shows a few examples of DNA sequences recognized by each custom detector, and their corresponding lists of tokens as provided by the recognizers.

Once the candidate sequences have been recognized and recorded, we move on to the next phase that we describe below.

### Phase 3: Automated Refinement and Post-Processing of the Recognized DNA Sequences

This phase is aimed at automatically refining and post-processing the sequences—i.e. lists of tokens—extracted during the previous activity. This includes: (1) discarding false positives, i.e. list of tokens that despite including strings belonging to ∑^+^, do not represent any sequences occurring in the paper—e.g. {"standard", "assay"—; (2) refining noisy sequences that include residual prefix or postfix expressions belonging to ∑^+^—e.g. {"ACGTACCCGTACGAT", "TAMRA", "T"—, and (3) splitting incorrectly merged sequences, which are composed of two or more different sequences linked by infix expressions belonging to ∑^+^—e.g. {"ACGTACCCGTACGAT", "and", "CGTACCGTACCAGGCTAC"}. Besides, the refined sequences—still represented as lists of tokens—need to be converted into singletons whose only element belongs to ∑^+^, a format suitable for the sequences to be used for querying the BLAST tool.

To address these issues, we have adopted a knowledge engineering approach. We have created a rule-based expert system [28] to automatically refine and post-process the extracted sequences. Table 3 shows the complete knowledge base, composed of eight rules, whereas Table 4 provides the description of functions, actions and symbols used by the different rules. Each rule was specifically designed to address a different issue. R1 is aimed at discarding sequences whose size—in terms of the number of symbols—is smaller than a predefined threshold *L_min_*. These sequences are unlikely to be true primer/probe sequences, and are thus discarded. We set the parameter *L_min _*to a size of seven symbols. On the other hand, R2, R3, R6 and R7 are aimed at refining noisy sequences by removing residual suffixes (rule R2) or prefixes (rule R3) and even English words incorrectly appended by the detectors either at the beginning (rule R6) or the end (rule R7) of a sequence. The key difference among the pairs of rules <R2, R3> and <R6, R7> is that <R2, R3> resorts to a list of problem affixes that can be composed of several tokens—e.g. {"TAMRA", "T")—whereas the <R6, R7> removes single words belonging to a custom English dictionary created by the authors and composed of words belonging to ∑^+^. Both the dictionary (see additional file 1: dictionary of problem English words) and the list of problem affixes (see additional file 2: list of problem affixes) are provided as additional material. By contrast, R4 aims to remove false positives—i.e. lists of tokens, all belonging to ∑^+^, that do not represent any real sequences. The rule resorts to the same dictionary used by rules R6 and R7 to discard all sequences—i.e. list of tokens—all of whose elements are in the dictionary. On the other hand, R5 is focused on splitting lists of tokens that contain two or more real sequences that were incorrectly merged by the detectors during the previous phase. This happens since two sequences may appear linked in the manuscript by words belonging to ∑^+^—e.g. the particle *and*—and thus the recognizers are unable to detect the end of the first sequence. To properly separate the sequences, the rule resorts to the list of affixes used by rules R2 and R3. Our approach can successfully separate incorrectly merged sequences composed of more than two real sequences by recursively applying rule R5. The aim of the last rule is to convert a sequence defined by a list of tokens into a singleton whose only element is the concatenation of all elements in the list—i.e. this is a post-processing rule. Rules are sorted in descending order of priority, R1 being the rule with highest priority.

**Table 3:** The knowledge base in a nutshell (I)

Rules	
**R1**	

	**IF **the sum of the lengths (i.e. number of characters) of all tokens *s_i _*belonging to *s *is smaller than *L*_min_, **THEN **discard *s*.

**R2**	∃*i *| *i *= affix_in_sequence_tails(*s*) → discard(*s*) ∧ add(*s*') *s*' = {*s*_1_,...,*s*_*i*-1_}

	**IF **an affix from the list of problem affixes is matched at the tail of s, **THEN **discard *s ***AND **add to the facts base a modified copy of *s *that does not include the tokens corresponding to the matched affix.

**R3**	∃*i *| *i *= affix_in_sequence_head(*s*) → discard(*s*) ∧ add(*s*') *s*' = {*s*_*i*+1_,...,*s_n_*}

	**IF **an affix from the list of problem affixes is matched at the head of s, **THEN **discard *s ***AND **add to the facts base a modified copy of *s *that does not include the tokens corresponding to the matched affix.

**R4**	

	**IF **all tokens *s_i _*from *s *belong to the custom dictionary of English words, **THEN **discard *s*.

**R5**	∃(*i*, *j*) | (*i*, *j*) = affix_within_sequence(*s*) → discard(*s*) ∧ add(*s*') ∧ add(*s'*') *s*' = {*s*_1_,...,*s*_*i*-1_}, *s'*' = {*s*_*i*+j_,...,*s*_*n*_}

	**IF **an affix from the list of problem affixes is matched within *s*, **THEN **discard *s ***AND **add to the facts base the sub-lists of tokens *s' *and *s'' *that include all the tokens in *s *that occur before and after the matched affix respectively.

**R6**	in_dictionary(s_1_) ∧ length(s_1_) ≥ 3 → discard(s) ∧ add(s') s' = {s_2_,...,s_n_}

	**IF **the first token of *s *belongs to the custom dictionary of English words **AND **its length (i.e. number of characters) is greater or equal to 3 **THEN **discard *s ***AND **add to the facts base the sub-list of tokens *s'*, that includes all but the first token of *s*.

**R7**	in_dictionary(*s_n_*) ∧ length(*s_n_*) ≥ 3 → discard(*s*) ∧ add(*s*') *s*' = {*s*_1_,...,*s*_*n*-1_}

	**IF **the last token of *s *belongs to the custom dictionary of English words **AND **its length (i.e. number of characters) is greater or equal to 3 **THEN **discard *s ***AND **add to the facts base the sub-list of tokens *s'*, that includes all but the last token of *s*.

**R8**	size(s) ≥ 2 → merge(s)

	**IF ***s *contains 2 or more tokens, **THEN **convert *s *into a singleton by concatenating all its tokens.

This table shows the complete knowledge base for refining DNA sequences. R1 is designed to discard short sequences. R2, R3, R5 and R6 are designed to refine noisy sequences, whereas R5 deals with incorrectly merged sequences. R4, by contrast, removes concatenations of dictionary words recognized by the detectors as valid sequences. Finally, R8 converts a list of tokens containing two or more elements into a singleton whose only element represents the refined sequence. The symbol *s *denotes a list of tokens *s *= {*s_1_*, *s_2_*,..., *s_n_*} of size *n*. See Table 4 for details on the functions, actions and symbols used by the different rules.

**Table 4:** The knowledge base in a nutshell (II)

**Functions, Actions and Symbols**

*S *= {*s*_1_, *s*_2_,...,*s_n_*} denotes a sequence (list of tokens) as recognized by the detectors during the second phase.

Length(*t*): function that returns the size—i.e. number of symbols—of a token *t *∈ ∑^+^

*L*_min_: minimum required size—i.e. number of symbols from ∑—for a sequence of tokens *s *not to be discarded. We set this parameter to 7 to enable the BLAST tool to produce results when queried using *s*.

discard(*s*): action that removes the sequence *s *from the facts base.

add(*s*): action that adds the sequence *s *to the facts base.

*L_A_*: list of problem affix expressions.

affix_in_sequence_tail(*s*): function that attempts to match all elements from *L_A _*to the tail of sequence *s*. If one or more matches occur, then the function returns the position of the first token belonging to the longest matched element. If there are two or more matches of the same length, then the one occurring first in the sequence is selected. For example, when processing the sequence of tokens {"TTACTCATGCCATACATAAATGGATA", "TAMRA", "T"}, the function would match the subsequence {"TAMRA", "T"} as the longest suffix belonging to *L_A _*occurring at the tail of *s*. Thus, the function would return the value 2, corresponding to the position of the token "TAMRA".

affix_in_sequence_head(*s*): function that attempts to match all elements from *L_A _*to the head of sequence *s*. If one or more matches occur, then the function returns the position of the last token of the longest matched element. If there are two or more matches of the same length, then the one occurring first in the sequence is selected. For instance, when processing the sequence of tokens {"and", "CTAGTTT", "ACGTAGGGT"}, the function would return the value 1, corresponding to the position of the token "and".

affix_within_sequence(*s*): function that attempts to match all elements from *L_A _*to a subsequence of *s*—excluding the head and the tail. If one or more matches occur, then the function returns a tuple <*p*, *s*>, where *p *denotes the position of the first token of the matched element and *s *is the size—i.e. number of tokens—of the match. If there are two or more matches of the same length, then the one occurring first in the sequence is selected. For example, when processing the list of tokens {"ACGTTTACGT", "TAMRA", "and", "CGATGGGA"}, the function would return the tuple (2, 1), corresponding to the subsequence {"TAMRA"}.

in_dictionary(*t*): function that searches the token *t *∈ ∑^+ ^in a custom dictionary created by the authors and composed of words belonging to ∑^+^. The function returns true if *t *is found in the dictionary and false otherwise.

size(*s*): function that returns the number of elements in the list of tokens *s*.

merge(*s*): function that returns the concatenation (preserving the original order) of all the elements in the lists of tokens *s*. For instance, if *s *= {"AAC", "TCG", "A"}, then merge(*s*) = {"AACTCGA"}.

To automatically refine a set of sequences extracted from a manuscript we proceed as follows: (1) all the sequences—i.e. lists of tokens—detected by the recognizers are added to the facts base, (2) the inference engine attempts to match the antecedents of the rules to the elements in the facts base, and (3) if there is one or more matches, then the rule with highest priority is fired. The execution of the rule changes the state of the facts base, and then the whole process is repeated from step 2 until no more rules can be fired. At the end of this process, the facts base contains only singletons—i.e. lists of tokens containing just one element—each being a valid sequence. For further details on the structure and functioning of rule-based inference engines, see [28].

Table 5 presents some examples of sequence refinement, including the initial contents of the facts base, the sequence of fired rules and the final state of the facts base. In these examples it is assumed that only one sequence is being processed at a time.

**Table 5:** Some examples of automated sequence refinement

List of Tokens	Execution Trace	Refined Singleton(s)
{"CATATTCACCTTTTCAGGCGTTTTGACCGT", "TAMRA", "T"}	<R2>	{"CATATTCACCTTTTCAGGCGTTTTGACCGT"}

{"ATAAC", "TCGAG", "GTGGA", "ATTCA", "TGGCA", "TCTAC", "TTCGT", "ATGAC", "TATTGC", "and", "AAGCT", "TGGTA", "CCTCA", "CTGCA", "GCAGA", "GCGCT", "GAGGC", "CCAGC", "AGCAC"}	<R5, R8, R8>	{"ATAACTCGAGGTGGAATTCATGGCATCTACTTCGTATGACTATTGC"}, {"AAGCTTGGTACCTCACTGCAGCAGAGCGCTGAGGCCCAGCAGCAC"}

{"than", "standard"}	<R4>	-

{"DNA"}	<R1>	-

{"TTCTTTTGGTGGACGATGTG", "and", "GAGGGACGC", "TTGGTAACG", "TAMRA", "and", "TCGCAAGCC", "AAGCAAATAC", "TAMRA", "T", "and", "GAGATAGGGTGCGATGGTTG", "TCGGCGATGACTACGACA"}	<R5, R3, R5, R5, R3, R8, R8, R8>	{"TTCTTTTGGTGGACGATGTG"}, {"GAGGGACGCTTGGTAACG"}, {"TCGCAAGCCAAGCAAATAC"}, {"GAGATAGGGTGCGATGGTTGTCGGCGATGACTACGACA"}

{"RNA", "strand"}	<R7, R1>	-

{":","GCGGCCTGATAAGGGATATTGGAAGC", "R", ":", "GGCGAAATTCATTAAAGAGGATCCTGACAC"}	<R3, R5>	{"GCGGCCTGATAAGGGATATTGGAAGC" }, {"GGCGAAATTCATTAAAGAGGATCCTGACAC" }

This table shows the results of using the knowledge-based system to refine some sample lists of tokens produced by different recognizers in phase 2. Each row of the table presents the refinement process of a single list of tokens, including: (1) the initial contents of the facts base, (2) the execution trace and (3) the final state of the facts base. All singletons in the facts base at the end of the execution are considered as valid and refined sequences.

Once the sequences provided by the detectors have been refined, we can proceed to the last activity described below.

### Phase 4: Linking the Recognized Sequences to their Corresponding Organism/Gene Information

In this phase, we use local copies of the nucleotide databases associated to two publicly available online resources—namely BLAST [29,30] and Entrez Nucleotide [31,32]—to connect the refined sequences to their corresponding organism and gene information, if available. To carry out this task, we created local instances of the BLAST-formatted database—downloaded from the NCBI website in FASTA format—and of the Entrez Nucleotide database in relational format. The latter was created using the BioPerl [33,34] package. The rationale for using local copies instead of the actual online resources is that users and tools performing massive queries of these online resources without express authorization may be delayed—and even banned—by the NCBI. Besides, if we query the Nucleotide database using a concrete GenBank Identifier (GI) via the provided web service, Nucleotide returns all available information about the matched record—sometimes over 4 MB—, whereas we are only interested in the organism/gene name. Instead, we retrieve just the required information from the local relational instance. The Nucleotide database was populated with data on micro-organisms only, since this method is aimed at detecting PCR primers and probes related to infectious diseases. However, the method can be easily adapted for other species simply by updating or replacing the database contents with the required data.

To obtain the organism/gene information for a specific paper, we use the BLAST software tool to query the BLAST-formatted database with all the detected sequences for the current manuscript. For each sequence, we select the best 10 matches provided by BLAST, and then we record (1) their GIs and (2) the relative positions of the query strings within the sequences associated with the matched GIs.

Once we have obtained the <GI, position> pair for each match, we query the relational instance of the Nucleotide database using the GI to retrieve the associated organism name. On the other hand, the position is used to find the specific location within the GI entry that contains the gene name(s), if available. For each processed manuscript, the output of this activity is a set of tuples <*paper*, *sequence*, *organism_name*, *gene_name*>.

**Figure 4:**
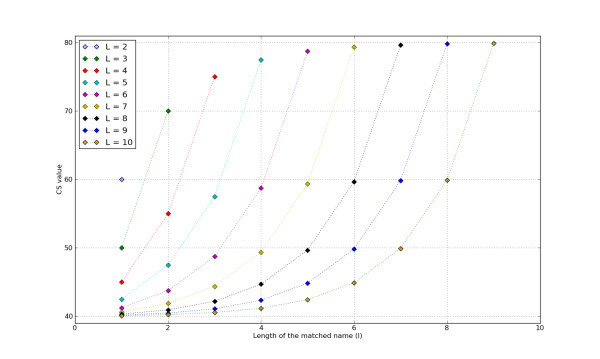
**Plot showing how CSs are assigned to the matched organism names depending on the length of the match**. Unlike regular English noun groups, where the meaning of the noun is narrowed by the preceding words, organism names are made more specific by post-positive words. The plot shows the CSs assigned to matches of length *l *for different values of *L*. This figure shows that the more specific—i.e. the longer—the matches are, the higher the assigned CS.

Once the sequences have been labeled with the information obtained from the BLAST-formatted and Nucleotide databases, then these results are checked against the text of the manuscript and automatically assigned a confidence score (CS) ranging from 0 to 100 points. The CS is assigned as follows. For each <*paper*, *sequence*, *organism_name*, *gene_name>*tuple, we search the *paper *for (1) all occurrences of the string *gene_name *and (2) different automatically generated spelling variants of the string *organism_name*—e.g. for "*Brucella Mellitensis 16M*" we would generate the variants "*Brucella Mellitensis*", "*Brucella*", "*B. Mellitensis 16M*" and "*B. Mellitensis*". Gene names are assigned a CS of 80 points if the string *gene_name *appears in the text, plus 20 additional points when the string *gene_name *co-occurs with the detected sequence in the same section. It is assigned a null CS otherwise. Conversely, the *organism_name *is assigned a score that depends on the size of the longest matched variant(s)—hereinafter denoted *l*—when compared to the full length *L *of the organism name provided by the Nucleotide database. The CS corresponding to a match—or matches—of length *l ≤ L *is calculated using the following function:

Figure 4 shows the plot corresponding to the CS_*L *_curves for 2 *≤ L ≤ *10. Each curve presents the assigned CS values for each allowed value of *l*. Besides, 20 additional points are added to the computed score if any of the matches of length *l *co-occurs in the same section with the sequence. An example of CS assignment follows. Let us suppose that the current sequence was labeled by the Nucleotide database with the organism name "*Brucella Mellitensis 16M*", thus *L *= 3. After searching the text for the different automatically generated strings we obtain matches for the following variants: "*Brucella*" (*l *= 1), "*Brucella Mellitensis*" (*l *= 2) and "*B. Mellitensis*" (*l *= 2), where "*B. Mellitensis*" appears in the same section as the detected sequence. Thus, the size of the longest match—matches in this example—is *2*. Therefore, the CS value is calculated as CS_*3*_(*2*) = 70. As one of the matches of length 2 co-occurs in the same section with the sequence, we add an extra 20 points to the calculated value. Thus, the final CS value for the organism name "*Brucella Mellitensis 16M*" related to the sequence *sequence *in the paper *paper *is 90—a value denoting high confidence in the reliability of the assigned organism name.

## Results

The software implementing the four-phase method for sequence extraction and annotation was developed using the Java programming language and the Apache PDFBox open source library [35]. The software, including both the binaries and the source code, can be freely downloaded from the project website. See the *Availability and requirements *section for further details.

To evaluate the performance of our method, a panel of experts composed of three senior molecular biologists from the Institute of Health Carlos III in Madrid (Spain) created a test set composed of 297 papers published in (1) several BMC journals (e.g. Virology Journal, BMC Microbiology or BMC Molecular Biology) and (2) different PLoS journals (e.g. PLoS One, PLoS Neglected Tropical Diseases or PLoS Genetics) containing 3999 primer and probe sequences. The papers in the test set were manually collected by the experts. They did not follow any specific criteria other than availability, provided that the manuscripts contained actual primer and/or probe sequences.

Experts were asked to manually analyze all the manuscripts in the test set to identify all valid DNA sequences occurring in the papers. They were also asked to specify which of the identified DNA sequences were actual primers and probes. According to the experts, less than the 2% of the DNA sequences occurring in the manuscripts were regular DNA sequences (i.e. not primers or probes), thus suggesting that papers reporting primers and probes rarely contain regular DNA sequences.

Each manuscript from the test set was fed through the method's pipeline to output a list of detected sequences—together with the context in which they appear within the documents—annotated with organism/gene information. These results were manually analyzed and assessed by the panel of experts.

Table 6 shows a summary of results of the sequence recognition and refinement activities. As shown in Table 6, our method achieved precision/recall rates of 97.98% and 95.77%, respectively. For a single PDF paper, our method required on average 397 milliseconds to complete the first three phases—i.e. 322 ms for phase 1 and 75 ms for phases 2 and 3—using a single PC equipped with an Intel^® ^Core™ 2 Quad Q6600 processor at 2.4 GHz and 2 GB RAM.

**Table 6:** Summary of results of the evaluation of activities 2 and 3

No. of sequences in the test set	Recognized (True Positives)	Not Recognized (False Negatives)	False Positives
3999	3830	169	79

**Precision**		97.98%	

**Recall**		95.77%	

**F-measure**		0.9686	

On the other hand, Table 7 summarizes the results of the evaluation of the annotation phase. As shown in Table 7, our method correctly annotated 76.66% of the 3830 detected primer/probe sequences. Conversely, 4.38% of the sequences were assigned incorrect organism names, whereas the Nucleotide database did not return any results for the remaining 18.96%. According to the experts, this happens because these are either *human *or *chicken *instead of *microorganism *sequences, and the local instance of the Nucleotide database currently contains information regarding microorganisms alone. Regarding the annotation with gene-related information of the 2936 sequences previously tagged with correct organism names, the Nucleotide database also returned 46.18% with correct gene names. The Nucleotide database did not return any results for the remaining 53.82%. Regarding performance issues, the annotation of all sequences appearing in a single manuscript took on average 15 minutes to complete.

**Table 7:** Summary of results of the assessment of activity 4

	Number of Sequences for Annotation	Correct Tagging	Incorrect Tagging	Information Not Found in the Nucleotide database
**Organism only**	3830	2936 76.66%	168 4.38%	726 671 *human* 55 *chicken* 18.96%

**Both organism and gene**	2936	1356 46.18%	0 0%	1580 53.82%

## Discussion

To our knowledge, the most recent biomedical text mining and information extraction research using scientific papers as a source of information does not accept manuscripts in PDF format as input. Instead these works resort to the plain-text or HTML versions of the documents, if available, or to tools, such as PDF to text/HTML converters or optical character recognition software, to extract the text to be processed from PDF files. However, these tools do not normally preserve the structure of the documents, and frequently fail to sort the sentences in the correct reading order. This hampers the information extraction and text mining activities. This problem is particularly serious when the target documents have a multiple column layout. To address these issues, our PDF-to-ST converter automatically creates a data structure that preserves the original organization of the document, including sentence order. This facilitates the text processing tasks, since the text corresponding to any section of the document can be easily retrieved from the ST data structure in the correct order. However, our approach requires a custom template to be created for each type of document to be processed—e.g. different journal layouts—to enable the PDF analyzer to properly build the ST structure. At the time of writing this paper these templates are created by manually inspecting the document layout. However, we are currently developing a tool to help users to create custom templates. We believe that both the ST data structure and the template creation tool are potentially valuable tools for biomedical informaticians working on text mining or information extraction and retrieval research.

Regarding the recognition and refinement of the primer/probe sequences present in the papers, our method achieves high precision and recall rates, as stated in the results section. In addition, the knowledge engineering approach we followed for sequence refinement successfully handles the different issues caused by the large number of English dictionary words belonging to ∑^+ ^that appear in the manuscripts. Our approach is also flexible, since the knowledge base will not normally have to be modified to refine sequences not currently being properly recognized—e.g. noisy sequences that the knowledge-based system fails to adequately refine due to words from ∑^+ ^that occur in the text but are missing from the dictionary or from the list of problem affixes—. Instead, the expert system can be easily adjusted by adding the required elements to the list of affixes or to the dictionary. This can be done manually or even automatically following an adapted relevance feedback-based approach [36]. Besides, the simplicity of the preliminary recognizers and the size of the knowledge base for sequence refinement—only eight rules—enables our method to achieve high throughput rates in sequence detection and refinement. Our method automatically recognizes sequences—with large precision/recall rates—as primers or probes, provided the system is fed with papers known to contain primer and probe sequences alone. The expansion of this feature to recognize and discriminate the different types of DNA sequences is a topic for future research. Besides, most major approaches for DNA/RNA sequence recognition in biomedical text focus on efficiently detecting—or aligning—sequences built upon the symbols A, C, G and T only [37-40]. On the other hand, the Kangaroo system [41] is a web-based pattern matcher that reports back all GenBank records that match a user query—i.e. a regular expression that may contain any of the symbols reported in Table 1. Conversely, our approach addresses a different issue, i.e. recognizes DNA sequences occurring in non-structured text.

Regarding the automated annotation of the detected sequences with their corresponding organism/gene information, the method assigns a valid organism name for most sequences (83.29%), as shown in Table 7. Conversely, for some sequences (15.45%), the method could not find any information in the database regarding the organism, since, according to the panel of experts, the sequences belonged to either *humans *or *chickens*. These results can be explained since the local copies of both the BLAST-formatted and Nucleotide databases were populated with microorganism-related information only. We did not load the information corresponding to other organisms since the method was initially designed to detect primers and probes related to microorganism-caused infectious diseases. However, we plan to populate the database with information related to other organisms in the near future. Regarding gene-related information, the system assigned correct gene names for 44.32% of the sequences that were properly annotated with their corresponding organism name. For the remaining sequences, the database did not provide any results. According to the panel of experts, this deficient gene-related information is due to the fact that the database does not contain this information, or even, that it is currently unknown. Regarding performance issues, annotating the sequences with their related information is computationally expensive, since multiple queries of different databases have to be launched. To address this issue, other distributed processing approaches may be helpful. This will enable the proposed pipeline to process a large number of documents in parallel, using multiple copies of the local databases.

## Conclusions

In this paper we present an original method for automatically extracting primer and probe sequences from scientific papers and annotating them with their corresponding organism/gene information. Our method can be used by biomedical researchers using molecular methods to diagnose and prescribe infectious diseases to facilitate tasks such as detecting the presence of a particular microorganism, or designing diagnostic PCR or microarrays. On the other hand, the extracted information can also be used to update the different existing primer/probe databases or to create a new data resource from the scratch. The proposed method can be extended to detect other types of biological sequences. In addition, the PDF-to-ST converter is a potentially valuable tool for different kinds of bioinformatics research using PDF files as a source of information.

## Availability and requirements

• **Basic implementation of the proposed method: **PrimerXtractor

• **Project website: **http://www.gib.fi.upm.es/en/PrimerXtractor

• **Operating systems: **Platform independent, tested on Windows Vista and Ubuntu Linux 9.10 (Karmic Koala).

• **Programming language: **Java.

• **Other requirements: **Java 1.6, MySQL Community Server 5, ActivePerl 5.8.8, BioSQL and BioPerl. See the online documentation at the project website for further details.

Documentation, source code (under GPL license) and binaries of PrimerXtractor are available in the project website. Other required software components are available at their corresponding sources.

## Authors' contributions

MGR participated in the creation of the method and in the design of the experiment, drafted the manuscript and supervised the work. AC participated in the creation of the method and in the design of the experiment, wrote the software tool implementing the presented method, and helped to draft the manuscript. VLA and GLC helped to draft the manuscript and led the evaluation process. GC, DDL, DPR, JC and FMS participated in the design of the experiment and helped to draft the manuscript. VM helped to draft the manuscript and to coordinate the work. All authors read and approved the final manuscript.

## Supplementary Material

Additional file 1 **Dictionary of problem English words**. list of English dictionary words belonging to ∑^+ ^used by the expert system for sequence refinement. Click here for file

Additional file 2 **List of problem affixes**. list of problem affixes used by the expert system for sequence refinement. Click here for file
